# The efficacy of three double-microencapsulation methods for preservation of probiotic bacteria

**DOI:** 10.1038/s41598-021-93263-z

**Published:** 2021-07-02

**Authors:** Pawiya Pupa, Prasert Apiwatsiri, Wandee Sirichokchatchawan, Nopadon Pirarat, Nongnuj Muangsin, Asad Ali Shah, Nuvee Prapasarakul

**Affiliations:** 1 Department of Microbiology, Faculty of Veterinary Science, Chulalongkorn University, Bangkok, 10330 Thailand; 2 College of Public Health Sciences, Chulalongkorn University (CPHS), Bangkok, Thailand; 3 Department of Pathology, Faculty of Veterinary Science, Chulalongkorn University, Bangkok, 10330 Thailand; 4 Department of Chemistry, Faculty of Science, Chulalongkorn University, Bangkok, 10330 Thailand; 5 Diagnosis and Monitoring Animal Pathogens Research Unit, Chulalongkorn University, Bangkok, 10330 Thailand

**Keywords:** Biotechnology, Microbiology

## Abstract

Lactic acid bacteria (LAB) are used as a probiotic alternative to antibiotics in livestock production. Microencapsulation technology is widely used for probiotic preservation. A variety of microencapsulation protocols have been proposed and compared based on chemicals and mechanical procedures. This study aimed to develop a double-encapsulated coating from alginate (1.5%) and chitosan (0.5%) by extrusion, emulsion, and spray drying methods using the LAB strains *Lactobacillus plantarum* strains 31F, 25F, 22F, *Pediococcus pentosaceus* 77F, and *P. acidilactici* 72N, and to monitor the basic probiotic properties of the encapsulated prototypes. The final products from each microencapsulation protocol were analysed for their appearance, probiotic properties and viable cell count. Using the spray drying method, particles smaller than 15 μm in diameter with a regular spherical shape were obtained, whereas the other methods produced larger (1.4–52 mm) and irregularly shaped microcapsules. After storage for 6 months at room temperature, the LAB viability of the spray-dried particles was the highest among the three methods. In all the LAB strains examined, the encapsulated LAB retained their probiotic properties in relation to acid-bile tolerance and antibacterial activity. This study highlights the efficacy of double-coating microencapsulation for preserving LAB properties and survival rate, and demonstrates its potential for probiotic application in livestock farms.

## Introduction

Probiotic bacteria are well-defined live micro-organisms which, when consumed in sufficient numbers, confer a beneficial health effect to the host’s gastrointestinal tract (GIT)^1^. In order to provide these benefits, probiotic bacteria must be presented at a minimum level of 10^6^ to 10^7^ colony forming units (CFU) per g or mL when they reach the gut^1–4^. Several stress factors during manufacturing, storage, and GIT transit are detrimental to the viability of probiotic bacteria^5–7^.

Microencapsulation coating of core material into capsules in the size range of micrometers up to millimeters is the most prominent technique to protect probiotic bacteria under adverse conditions and improve their survival at the final destination^8–11^. Alginate has often been used in microencapsulation procedures because it is simple to manipulate, compatible with almost all encapsulation methods, low-cost, and is a non-toxic material^12,13^. However, alginate microcapsules readily disintegrate in highly acidic environments, as occurs in the stomach^14–16^. To decrease these drawbacks, good biofilm-forming materials, such as chitosan, have been chosen for coating alginate beads to enhance their protection^15,17,18^. Various methods have been widely used for probiotic cell encapsulation, including extrusion, emulsion, and spray drying^12,19^. Production of stable microparticles of a low diameter and homogeneous size distribution is an advantage of the spray drying method compared to the extrusion or emulsion methods. Nonetheless, this method can reduce the cell vitality due to dehydration and thermal inactivation of the probiotic cells during the process^12,20–22^. There has been relatively little comparison of the efficiency and viability of these different encapsulation methods for probiotic cells. Moreover, there is no evidence to confirm the persistence of probiotic properties post-encapsulation.

This study aimed to evaluate the efficacies of three microencapsulation methods (extrusion, emulsion, and spray drying) on the viability, thermotolerance, encapsulation yield (EY) percentage, and probiotic properties (acid-bile tolerance and antimicrobial activity) of LAB strains double-coated with alginate-chitosan.

## Results

### Size and morphology of microcapsules

**Figure 1:**
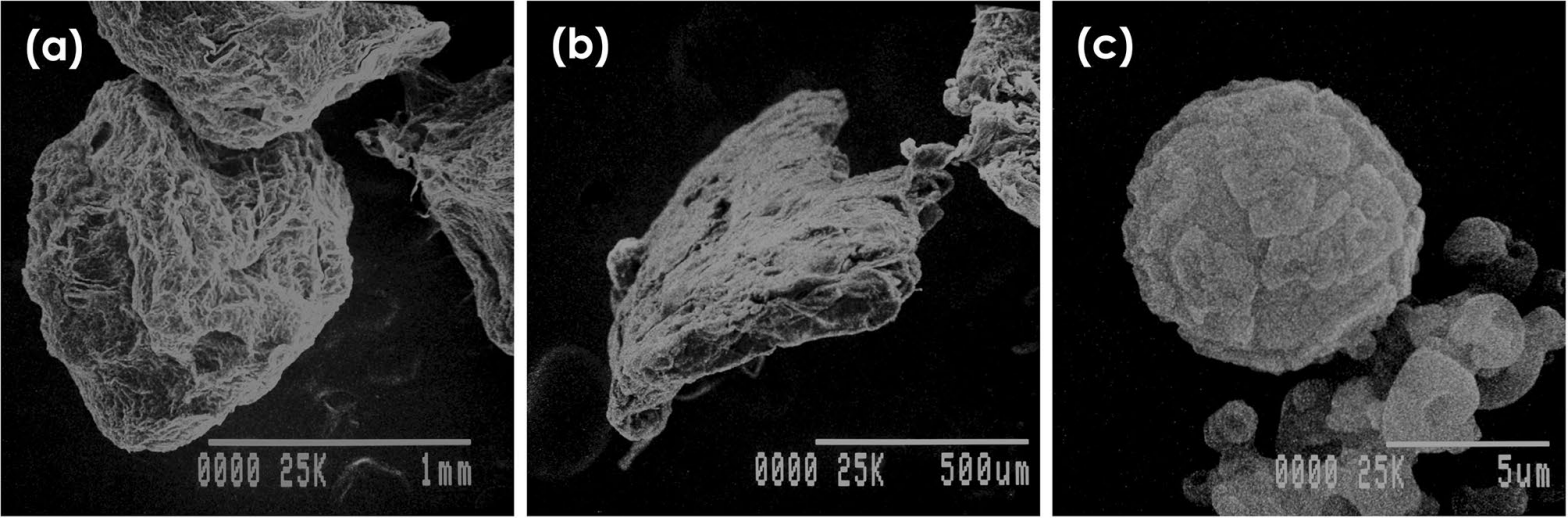
Representative SEM images of alginate-chitosan microcapsules of L22F (as a representative example of the five LAB strains) prepared by the (**a**) extrusion, (**b**) emulsion, and (**c**) spray drying methods.

From the SEM analysis, the size of the encapsulated products among the three methods were significantly (*P* < 0.05) different, and their shapes varied, but within each method they were uniform (Table 1 and Fig. 1). The microcapsules obtained by the extrusion method were droplet-like particles with a mean diameter of approximately 1.5 mm. Those formed by emulsion were tiny flake shaped (0.5 mm in diameter), whereas those from the spray drying method were spherical powdery particles with a mean diameter of less than 15 μm. For each method, there was no difference in the size (Table 1) or morphology (not shown) of the particles between the five LAB strains.

**Table 1:** Size and EY of the microcapsules. The different lowercase letters within each column indicate significant differences between methods (*P* < 0.05).

Method	Size of microcapsule (μm)					EY (%)				
	L22F	L25F	L31F	P77F	P72N	L22F	L25F	L31F	P77F	P72N
Extrusion	1516 ± 304.86^c^	1469 ± 273.51^c^	1429 ± 412.11^c^	1504 ± 282.42^c^	1443 ± 370.89^c^	93.52 ± 0.11^b^	93.49 ± 0.34^b^	93.22 ± 0.17^b^	93.11 ± 0.08^b^	93.39 ± 0.32^b^
Emulsion	482.11 ± 58.59^b^	497.11 ± 67.51^b^	527.75 ± 57.69^b^	495.75 ± 70.04^b^	524.38 ± 67.56^b^	94.10 ± 0.10^b^	93.55 ± 0.21^b^	93.27 ± 0.63^b^	92.66 ± 0.05^b^	93.83 ± 0.21^b^
Spray dry	12.74 ± 1.710^a^	13.10 ± 1.83^a^	12.90 ± 2.02^a^	12.58 ± 1.74^a^	13.10 ± 3.35^a^	74.56 ± 0.11^a^	74.50 ± 0.02^a^	74.67 ± 0.27^a^	73.64 ± 0.09^a^	75.03 ± 0.05^a^
*P*-value	< 0.05	< 0.05	< 0.05	< 0.05	< 0.05	< 0.05	< 0.05	< 0.05	< 0.05	< 0.05

### Efficacy of microencapsulation

#### Encapsulation yield (EY)

The EY rates were found to range from 73.64 to 94.10% (Table 1). The EY was higher in the extrusion and emulsion methods (at 93% approximately) than in the spray drying method (*P* < 0.05), but there was no significant difference between different LAB strains.

#### Viability test

**Figure 2:**
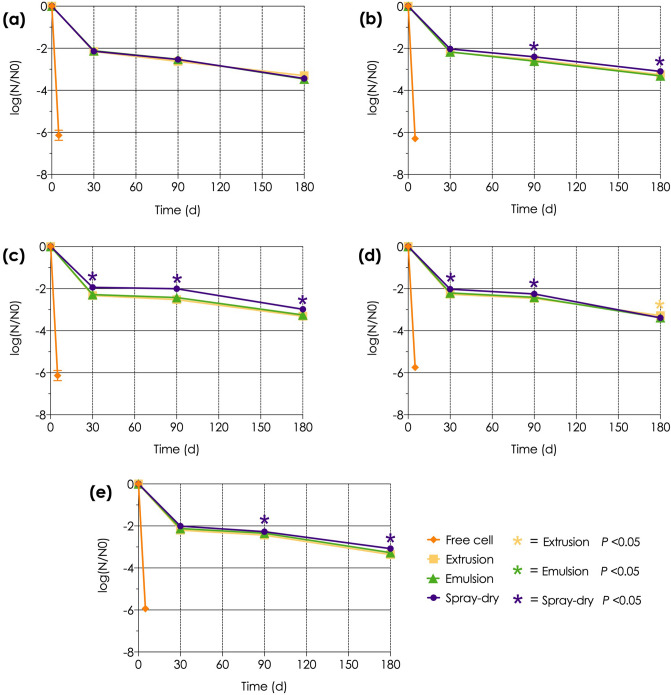
Comparison of the survival level between the non-capsulated and differently encapsulated LAB (**a**) L22F, (**b**) L25F (**c**) L31F, (**d**) P77F and (**e**) P72N strains over 6 months storage at room temperature. The asterisks represent statistically significant differences (*P* < 0.05). The yellow, green, and purple asterisks represent the significance of differences for the extrusion, emulsion, and spray-drying methods, respectively.

The viability of the microencapsulated LAB obtained from all three methods were significantly (*P* < 0.05) different from the free cells (Fig. 2). The alginate-chitosan coated LAB showed a greater viable cell count during the 6-months storage at room temperature (25–28 °C) than the others. For all three microencapsulated methods, the number of viable cells was reduced by about 2.0 log CFU/g after 1-month storage but by less than 3.5 log CFU/g after 6 months. Meanwhile, the free LAB cells could not be grown after storage for only 3 days.

#### Thermotolerance test

**Figure 3:**
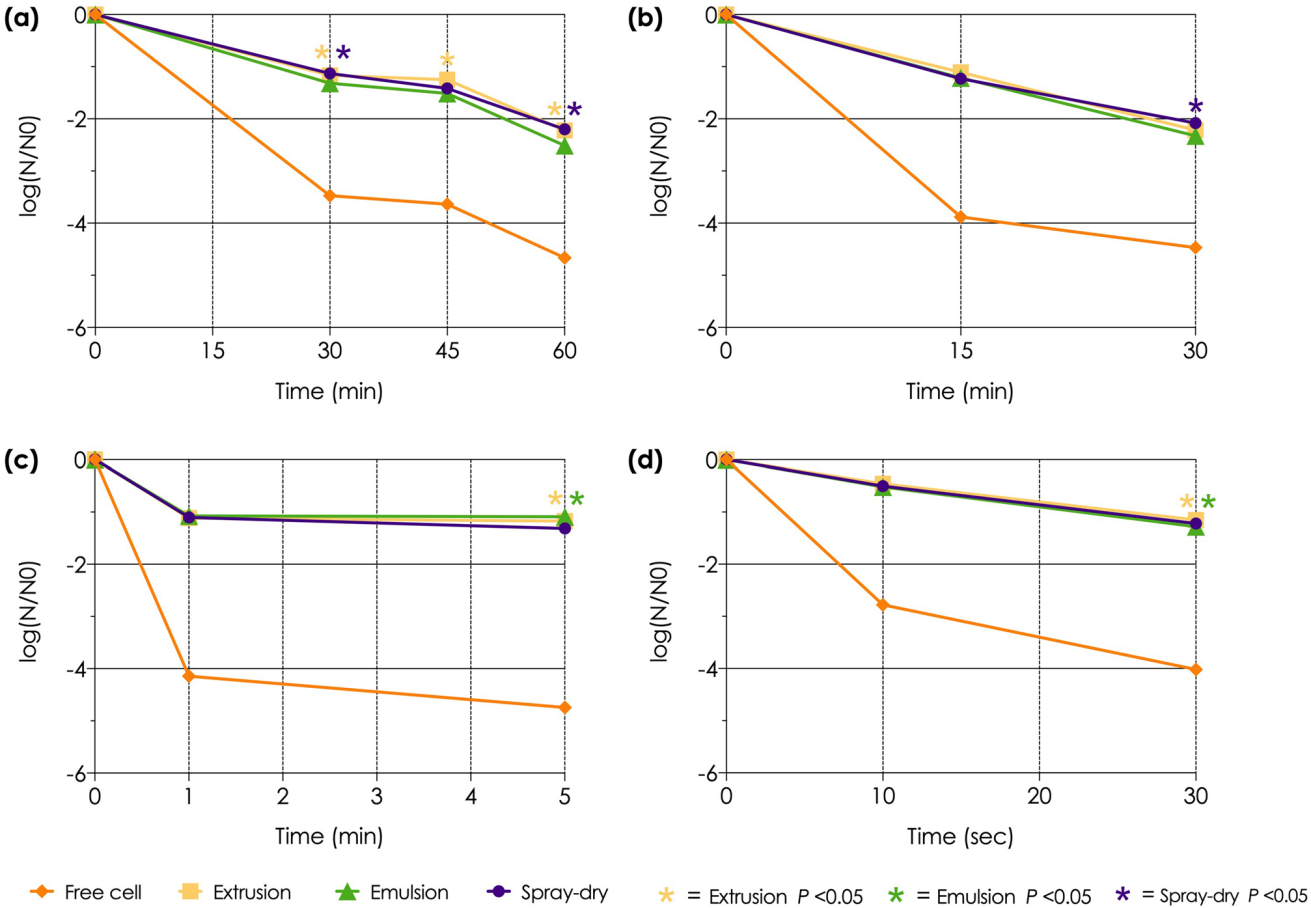
Comparison of survival between the non-capsulated and differently encapsulated L22F strains after heat treatment at (**a**) 60 °C, (**b**) 70 °C (**c**) 80 °C, and (**d**) 100 °C. The spray drying procedures were performed under an inlet temperature of 130 °C. The asterisks represent statistically significant differences (*P* < 0.05). The yellow, green, and purple asterisks represent the significance of the extrusion, emulsion, and spray-drying methods, respectively.

LAB microencapsulated by each of the three methods tolerated high-temperature treatment significantly (*P* < 0.05) better than did the free cells (Fig. 3). For all three microencapsulation methods, the number of viable cells was reduced by approximately 2.5 log CFU/g after 60 °C for 60 min and 70 °C for 30 min, compared to about 1.5 log CFU/g after 80 °C for 5 min and 100 °C for 30 s.

### Confirmation of probiotic properties after encapsulation

#### Acid and bile tolerance test

After 6 months storage, all five tested strains of LAB encapsulated by the three different methods still showed a similar tolerance (viability) to acid and bile adjusted MRS broth compared to the fresh cultures of LAB strains (Table 2).

**Table 2:** Viable colony count of live-cultures and 6-month stored encapsulated LAB after acid-bile incubation. Colony counts ≥ 10^4^ CFU/mL indicate tolerance to acid and bile.

Method	Colony count									
	HCl adjusted MRS					Oxgall adjusted MRS				
	L22F	L25F	L31F	P77F	P72N	L22F	L25F	L31F	P77F	P72N
Live culture	1.10 × 10^5^ ± 1.41	0.43 × 10^5^ ± 1.26	0.98 × 10^5^ ± 1.71	0.17 × 10^5^ ± 0.42	2.08 × 10^5^ ± 1.26	1.33 × 10^4^ ± 4.11	1.25 × 10^4^ ± 1.30	1.48 × 10^4^ ± 3.60	1.08 × 10^4^ ± 2.50	2.28 × 10^4^ ± 3.95
Extrusion	1.10 × 10^5^ ± 0.81	0.48 × 10^5^ ± 2.36	0.80 × 10^5^ ± 2.58	0.17 × 10^5^ ± 0.38	1.88 × 10^5^ ± 1.71	1.23 × 10^4^ ± 3.70	1.10 × 10^4^ ± 3.56	1.50 × 10^4^ ± 5.66	1.09 × 10^4^ ± 3.11	2.13 × 10^4^ ± 1.71
Emulsion	1.13 × 10^5^ ± 1.70	0.77 × 10^5^ ± 0.50	0.88 × 10^5^ ± 1.71	0.17 × 10^5^ ± 0.10	2.08 × 10^5^ ± 1.26	1.40 × 10^4^ ± 4.16	1.23 × 10^4^ ± 3.40	1.60 × 10^4^ ± 2.16	1.03 × 10^4^ ± 2.22	2.25 × 10^4^ ± 1.30
Spray dry	1.03 × 10^5^ ± 1.50	0.23 × 10^5^ ± 2.31	0.95 × 10^5^ ± 1.73	0.15 × 10^5^ ± 0.13	1.93 × 10^5^ ± 2.1	1.00 × 10^4^ ± 0.82	1.10 × 10^4^ ± 1.83	1.70 × 10^4^ ± 1.83	1.05 × 10^4^ ± 4.04	2.43 × 10^4^ ± 2.62

#### Antibacterial effect

After 6 months of storage, the encapsulated LAB still retained an antibacterial ability against pathogenic bacteria that ranged from an intermediate to a strong inhibition level relative to the live culture form (Table 3). However, LAB strains L31F and L22F showed a weaker inhibition to ETEC and EHEC after 180 days of storage when the microcapsules were formed by the emulsion or extrusion methods.

**Table 3:** Antibacterial activity of the cell free supernatant (CFS)* of live-cultured and 6-month stored encapsulated LAB. *Non-neutralized CFS; The diameters of the inhibition zones were measured and interpreted as (+) weak inhibition (6–9 mm), (++) intermediate inhibition (10–13 mm), (+++) strong inhibition (14–16 mm) and (++++) very strong inhibition (> 17 mm).

Method	ETEC					EHEC					*Salmonella choleraesuis*				
	L22F	L25F	L31F	P77F	P72N	L22F	L25F	L31F	P77F	P72N	L22F	L25F	L31F	P77F	P72N
Live culture	+++	++	+++	++	++	+++	++	+++	++	+++	+++	+++	+++	+++	+++
Extrusion	+++	++	++	++	++	+++	++	++	++	+++	+++	+++	+++	+++	+++
Emulsion	++	++	++	++	++	+++	++	++	++	++	+++	+++	+++	+++	+++
Spray dry	+++	++	+++	++	++	+++	++	+++	++	+++	+++	+++	+++	+++	+++

## Discussion

In this study, the double-coated bead size formed by extrusion and spray drying resembled those reported previously^14,23^, whereas those formed by the emulsion method were larger than previously reported^24,25^. In previous studies, extrusion beads over 100 μm in size did not enhance nutrient availability or reduce the palatability of the consolidated food product^26,27^. From size alone, the alginate-chitosan powder obtained from the spray drying method would be the most suitable to apply for delivery to hosts via feed or water. Thus, encapsulation by spray drying could transform free LAB cells into a usable powdery form that could be applicable for combining in a livestock meal.

The lower EY rate obtained by spray drying could be of concern in a scaled-up process, but is still in the acceptable range and consistent with that previously reported^28^. The high EY rates of 97.4–99.9% that were obtained with the extrusion or emulsion methods were concordant with previous studies^14,24,25^. The higher EY obtained by the extrusion and emulsion methods were due to the use of a lower temperature and less osmotic stress in the process compared to spray drying^6,20,29^. To improve the EY in the process, it has been suggested that increasing the number of live probiotic cells, supplementing them with certain protective agents (such as maltodextrin, inulin, reconstituted skimmed milk, or low melting point fat), or reducing the inlet temperature during the drying step could be used^20,30^.

Although the results of this study were comparable to findings with emulsion-encapsulation of *Bifidobacterium animalis* BB-12 during storage for 90 days^31^, they contrasted with those of another study where the viability of microencapsulated *L. lactis* formed by extrusion showed a reduction in viability of up to 5 log CFU/g after only 30 days in storage^32^. Single alginate coated probiotic bacteria are fragile and tend to lose viability more than double-coated ones^17^. Therefore, the double coating of alginate beads with chitosan, as used in this study, could be recommended to protect the viability of LAB for at least 6 months at room temperature. In contrast, the viability of *L. acidophilus* La-5 and *L. rhamnosus* B442 have been reported to decrease by only 0.43–0.62 log CFU/g after storage for 32–90 days when prepared by the spray dry method^26,28^. However, those studies maintained the moisture content of the spray-dried products at 1.8–4.79% during storage, whereas in the current study the products were not subjected to moisture control: this might have impacted on the viable cell count since the survival of encapsulated probiotics after spray drying is related to the water content^33,34^. To promote LAB viability, the water content should be reduced, and the storage temperature kept at 4 °C^35–37^. Nevertheless, after six months of storage, the LAB double-coated by the spray drying method in the current study exhibited a better (lower) logarithmic reduction in the viable cell count than those formed by the extrusion or emulsion methods for all five tested LAB strains. In contrast, in another study the viability of *L. casei* encapsulated with alginate-whey protein by the emulsion method was reported to be higher by 0.8 log CFU/g than with the spray dry method coating with alginate-chitosan after three months of storage at 4 °C^22^. These differences may be due to the different properties of the encapsulating materials applied. In contrast to the stability of the viable cell count at room temperature over 180 days of storage found in the current study, the loss in cell viability during the initial spray dry method was clear, and in accord with previous studies that found that spray-dried products can be stored for prolonged periods^20,34,38^.

The reductions in the microencapsulated LAB count after heat treatment (60 °C for 60 min, 70 °C for 30 min, 80 °C for 5 min, and 100 °C for 30 s) were less than 2.5 log CFU/g. In contrast, it has been previously reported that the viability of *L. rhamnosus* GG double-coated with alginate-chitosan by the external gelation method was reduced by 5.9, 7.4, 6.5 and 3.3 log CFU/mL after incubating at 60 °C for 30 min, 70 °C for 30 min, 80 °C for 1 min, or 100 °C briefly, respectively^39^. Therefore, it seems probable that the extrusion, emulsion, and spray drying methods used in the current study gave a better thermos-resistance performance (higher LAB cell viability) than would have been achieved with the alternative external gelation method. Furthermore, at 60 and 70 °C, the L22F strain that was double-coated by the emulsion or spray drying methods showed a higher viability than was achieved with the extrusion method. In contrast, the extrusion and emulsion double-coated L22F cells exhibited a higher viability after the 80 and 100 °C treatments than did the spray-dried L22F. Interestingly, previous studies showed a rather dissimilar trend. *L. casei* NCDC-298 encapsulated with alginate by the emulsion method at 60 °C for 20 min was reported to have a reduction in cell viability of 2–3 log CFU/g^40^, which was lower than with chitosan-coated alginate-starch LAB formed by the extrusion method that had a 1.42 log CFU/mL reduction after heat treatment at 60 °C for 30 min^41^. The results of the current study suggest that the methods used might be useful for industrial processing, such as feed pellet production that requires at least 80 °C for 17 s to form pellets. In addition, the encapsulation could prolong the viability of LAB by protecting from temperature fluctuations during storage in livestock farms.

Under long-term storage, LAB-containing microcapsules might be exposed to various stresses, such as starvation, heat, osmosis, or oxidation. Such stresses cause LAB to shift their physiological state by reducing the synthesis of DNA and proteins, and finally they may lose some functionality^42,43^. In this study, all five LAB strains still maintained their acid and bile tolerance, although the degree of such was specific to each LAB strain^43^.

Previously, the antibacterial properties of *P. acidilactici* UL5, *L. reuteri*, and *L. salivarius* were found to decline after encapsulation and prolonged storage^44^. This may be explained by metabolic changes following carbohydrate starvation that reduced synthesis of metabolic end products^42,43^. Moreover, active antimicrobial substances (bacteriocins or antimicrobial peptides) secreted by LAB strains may remain trapped inside microcapsules, which could reduce the amount of these active substances in cell free supernatants (CFS) and lower the apparent antibacterial effect. The results of this study indicated that the emulsion and extrusion methods slightly reduced the antibacterial ability of LAB, although this depended on the LAB strain and the target pathogenic bacteria. Nevertheless, all three encapsulation methods still showed a satisfactory outcome in preserving antibacterial activity, which might be applicable for industrial processes and livestock farm usage.

## Conclusion

For alginate-chitosan double-coated LAB, this study revealed that three methods of encapsulation (extrusion, emulsion, and spray drying) could provide a high final bacterial concentration. The spray dry method gave smaller diameter sized particles as an easily handled powder for mixing with feed product. The alginate-chitosan beads from all three methods significantly improved the survival of LAB over 6-month storage at room temperature and after high-temperature treatment. Moreover, the six-month stored encapsulated LAB still retained their probiotic properties of acid-bile tolerance and antibacterial ability. Therefore, the use of microencapsulation by the spray drying method is strongly recommended for probiotic preparation for livestock farms.

## Materials and methods

### Bacterial strains and culture condition

The five strains of LAB used in this study included *Lactobacillus plantarum* 31F (L31F), *L. plantarum* 25F (L25F), *L. plantarum* 22F (L22F), *Pediococcus pentosaceus* 77F (P77F), and *Pediococcus acidilactici* 72N (P72N), and were selected since they have been reported to have a high potential as swine probiotic strains^45–47^. The strains were maintained in de Man Rogosa Sharpe (MRS) broth (Becton, Dickinson and Company, Maryland, USA) containing 20% (v/v) glycerol at − 80 °C. Frozen stocks were cultured in MRS broth and incubated at 37 °C for 18–20 h. Each LAB strain was harvested by centrifugation at 3000*g* for 10 min at 4 °C and then washed twice in 0.85% (w/v) saline solution. The cell pellets were resuspended in normal saline and prepared at a final concentration of 10^9^ CFU/mL^48^. The cell suspension of each LAB strain was divided into four groups; free cells (control) and three methods of microencapsulation.

### Microencapsulation and coating procedures

#### Internal coating step

A 1.5% (w/v) alginate solution (Sigma-Aldrich, Missouri, USA) was initially prepared for inner encapsulation. Initially, 9 log CFU/mL of the respective LAB strain was mixed with 20 mL of alginate solution at a 1:5 (v/v) ratio and was then ready for the further microencapsulation step^14^. For the extrusion method, the alginate mixtures were added dropwise through a 3 mL-syringe into 100 mL of CaCl_2_ (1 mol/L) (Merck KGaA, Darmstadt, Germany) and left for 30 min for gelation to achieve the alginate beads^39,49^. For the emulsion method, the alginate beads were settled by adding the alginate mixtures to 100 mL of soybean oil (Sigma-Aldrich, Missouri, USA) containing 0.2% (v/v) of Tween 80 while stirring with a magnetic stirrer. Next, 100 mL of CaCl_2_ (1 mol/L) was added into the mixture to solidify the alginate beads, which were then harvested by centrifugation at 350*g* for 10 min at 4 °C^40^. Alginate beads obtained from the extrusion and emulsion methods were rinsed with and then kept in 0.1% (w/v) peptone water (Becton, Dickinson and Company, Maryland, USA) at 4 °C^14^. For the spray drying method, alginate mixtures were atomized through the spray dryer (Mini Spray Dryer B-290, Buchi, Flawil, Switzerland) with an inlet temperature of 130 °C, and the alginate beads were then collected from the collecting vessel^23^.

#### External coating step

A 0.5% (w/v) chitosan solution (Union Chemical 1986, Bangkok, Thailand) in 100 mL of 1% (v/v) acetic acid (Merck KGaA, Darmstadt, Germany) was prepared for the outer microcapsule coating, and the solution was filtered through a nylon cloth to separate the insoluble material^17^. The alginate beads obtained from the extrusion and emulsion methods were immersed into the chitosan solution and shaken at 100 rpm for 40 min. The obtained alginate-chitosan coated beads were then washed with and stored in 0.1% (w/v) peptone water at room temperature^14,49^. Meanwhile, 1 g of the beads obtained from the spray drying method was added to 100 mL of the above 0.5% (w/v) chitosan solution and then atomized through a spray dryer. The alginate-chitosan double-coated beads were then collected from the collecting vessel and kept at room temperature^23^.

### Size and morphology of the microcapsules

The size and morphology of the obtained probiotic microcapsules were examined using light microscopy and scanning electron microscopy (SEM). The capsules were placed on a specimen aluminum stub with the help of double-sided sticky tape and coated in a sputter coater for 2 min at an accelerating voltage of 15 kV^24,25^.

### Efficacy evaluation of the microencapsulations

#### The EY

Calculation of the EY percentage was modified from that previously reported^28^ as follows: Eq. (1), where N_0_ and N represented the number of viable CFUs before and after the encapsulation process, respectively.

1 $${\text{EY }} = {\text{ }}\left( {{\text{logN}}/{\text{logN}}_{0} } \right){\text{ }} \times {\text{ 1}}00~.$$

#### Viability test

The obtained microencapsulated products from the three methods, along with free cells, were stored at room temperature for up to 6 months. Evaluation of the LAB viability was performed at 0, 30, 90, and 180 days after encapsulation. For decapsulation, 1 g of the respective alginate-chitosan coated bead preparation obtained from the extrusion or emulsion method was liquefied in 9 mL sodium citrate (0.1 mol/L, pH 6.0) (Merck KGaA, Darmstadt, Germany) and stirred for 10 min^8^. Meanwhile, 1 g of the obtained spray-dried beads and 1 mL of free cells were suspended in 9 mL of 0.1% (w/v) peptone water^23^. The liquefied suspensions were decimally diluted in 0.1% (w/v) peptone water and plated on MRS agar by the drop plate method. Viable cells were enumerated as the number of colonies after incubation at 37 °C for 48 h and converted to CFU/mL or g^50^. The reduction in viability was characterized in terms of the log (N/N_0_), where N_0_ and N represented the initial and actual viable CFUs, respectively^39^.

#### Thermotolerance test

For the thermal-tolerance evaluation, 1 mL of preheated 0.1% (w/v) peptone water at 60, 70, 80, and 100 °C was mixed with 1 g of alginate-chitosan coated beads or 1 mL of free cells, and then incubated at that temperature set for various times as follows: 60 °C for 30, 45, and 60 min; 70 °C for 15 and 30 min; 80 °C for 1 and 5 min; and 100 °C for 10 and 30 s. The double-coated beads were decapsulated, and their cell viability was determined as described previously^8,23,39,50^.

### Confirmation of probiotic properties after encapsulation

#### Acid and bile tolerance test

The probiotic properties were determined as previously reported^46,51^. After six months storage, 1 g of alginate-chitosan coated beads obtained from the extrusion, emulsion, or spray drying methods were decapsulated as described previously^8,23^. Then 10 µL of the liquefied suspension was cultivated on MRS agar at 37 °C for 18–24 h. Fresh LAB strains were used as control (100% viability). A full loop of LAB cells was inoculated into the MRS broth and incubated at 37 °C for 24 h. The overnight cultures of microencapsulated products and fresh cells were harvested by centrifugation at 10,000*g* for 10 min at 4 °C.

For the acid tolerance test, overnight cultures at approximately 1 × 10^8^ CFU/mL were re-suspended in 1 equivalent/L MRS broth adjusted to pH 2.0 with HCl (Carlo Erba Reagents, Val de Reuil, France). For the bile tolerance determination, 1 × 10^8^ CFU/mL of the cultures were inoculated in 0.3% (w/v) Oxgall powder (Sigma-Aldrich, Missouri, USA) supplemented MRS broth (pH 6.5). After incubation at 37 °C for 12 h, viable bacterial counts were measured using the spread plate method. The culture suspension was diluted serially and plated on MRS agar, then enumerated after incubation at 37 °C for 48 h. Microencapsulated products with ≥ 10^4^ CFU/mL indicated the persistency of acid-bile tolerance.

#### Antibacterial effect

Evaluation of the antibacterial activity was performed using the agar well diffusion assay as reported^45,52^. In brief, 1 g of 6-month stored alginate-chitosan coated beads prepared by the three methods were decapsulated and prepared as an overnight culture as described in the previous section “Acid and bile tolerance test”. Likewise, acid and bile tolerance tests were also performed from frozen stocks (− 80 °C) of the respective free cell LAB. Each overnight culture was centrifuged at 7000*g* for 5 min at 4 °C, and the supernatant was harvested and filtered through a sterile filter (0.22 μm pore-size) (Corning, New York, USA) to achieve a cell free supernatant (CFS).

Three strains of indicator pathogens, comprised of enterotoxigenic *E. coli* (ETEC) VP10Ltb + , enterohaemorrhagic *E. coli* (EHEC) T2R2-26-HB2, and *Salmonella choleraesuis* 86-1, were grown at 37 °C overnight on Luria–Bertani (LB) broth (Becton, Dickinson and Company, Maryland, USA) at 10^8^ CFU/mL. Two mL of each indicator pathogen were thoroughly mixed with 18 mL of LB agar [1.2% (w/v), 45 °C] and poured into a Petri-dish. The agar was left for 30 min, and then 6-mm diameter wells were punched with a sterile tip. Thereafter 50 µL of the respective CFS was added into each well. In addition, each CFS was also adjusted to pH 7.0 with 4 mol/L NaOH (pH 7.0) (Carlo Erba Reagents, Val de Reuil, France) and likewise assayed, to rule out an acidic effect^45^. The plates were incubated at 37 °C for 24–48 h, and the antibacterial activity was recorded as the growth-free inhibition zone around each well. Inhibition zones were measured from the edge of each well. The CFS from the free cell LAB and MRS medium without an inoculating strain were used as positive and negative controls, respectively.

### Data analysis

Numerical data were presented as the mean ± standard deviation (SD) of replicated determinations. The average microcapsule diameter, EY percentage, log reduction of viability, and viable bacterial cell count among the three different methods (extrusion, emulsion, and spray drying) were compared. All of the experiments were performed in triplicate. Those parameters were analyzed by one-way ANOVA, and comparison of means were tested by Tukey’s multiple range tests using the SPSS version 22.0 software (IBM, NY, USA). The effects were considered to be significant at *P* < 0.05.
